# Limb Girdle Muscular Dystrophy Type 2E Due to a Novel Large Deletion in SGCB Gene

**Published:** 2017

**Authors:** Soudeh GHAFOURI-FARD, Feyzollah HASHEMI-GORJI, Majid FARDAEI, Mohammad MIRYOUNESI

**Affiliations:** 1 Department of Medical Genetics, Shahid Beheshti University of Medical sciences, Tehran, Iran; 2 Genomic Research Center, Shahid Beheshti University of Medical Sciences, Tehran, Iran; 3 Department of Medical Genetics, Shiraz University of Medical Sciences, Shiraz, Iran

**Keywords:** Limb-girdle muscular dystrophy, Mutation, SGCB

## Abstract

Autosomal recessive limb-girdle muscular dystrophies (LGMD type 2) are a group of clinically and genetically heterogeneous diseases with the main characteristics of weakness and wasting of the pelvic and shoulder girdle muscles. Among them are sarcoglycanopathies caused by mutations in at least four genes named SGCA, SGCB, SGCG and SGCD. Here we report a consanguineous Iranian family with two children affected with LGMD type 2E.

Mutation analysis revealed a novel homozygous exon 2 deletion of SGCB gene in the patients with the parents being heterozygous for this deletion. This result presents a novel underlying genetic mechanism for LGMD type 2E.

## Introduction

Autosomal recessive limb-girdle muscular dystrophies (LGMD type 2) comprise a group of clinically and genetically heterogeneous diseases with the main characteristics of weakness and wasting of the pelvic and shoulder girdle muscles (1). As various LGMD subtypes are difficult to be distinguished merely by their clinical manifestations, a combination of immunohistochemical and immunoblot analyses, and subsequent DNA sequencing have been suggested for identification of the underlying causes (2). 

Based on the former method, LGMD can be classified to sarcoglycanopathy, calpainopathy, dysferlinopathy, and dystroglycanopathy subtypes (3). 

Sarcoglycan complex in skeletal muscle is made by the protein products of four genes namely SGCA, SGCB, SGCG and SGCD. This complex contributes to the stability of dystrophin-dystroglycan complex as well as the stability of the plasma membrane cytoskeleton (4). Overall, mutations in 16 genes have been associated with different types of LGMD (5). Severe childhood-onset LGMD is mostly associated with the mutations of SGCG, SGCA, SGCB or SGCD genes (LGMD2C-2F, respectively) (6). LGMD2E (MIM# 604286) has been a clinically heterogeneous disorder associated with both truncating and missense mutations in SGCB gene as well as large homozygous microdeletion of chromosome 4q11-q12 that comprises the whole SGCB gene (7).

**Fig 1:**
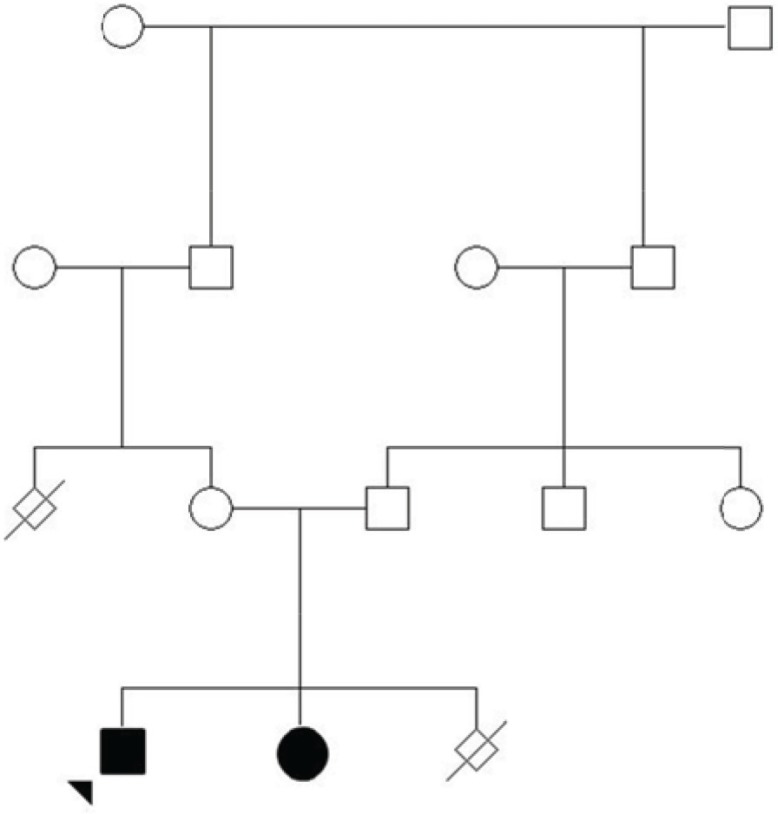
The family pedigree

Case study Here we report a consanguineous Iranian family with two children (12 and 7 yr old) affected with LGMD (Figure 1). Both siblings suffered from progressive muscle weakness with clinical onset of an age of six years. 

No cardiac abnormalities were reported in their electrocardiography or echocardiography. The older sib became wheelchair-dependent by the age of 10 yr. Their mental development was normal. Creatine kinase (CK) levels were elevated to 100 times of normal values, whereas lactate dehydrogenase (LDH) levels were up to 10 times of normal range. A slight elevation in hepatic enzyme levels was also indicated.

The electromyogram showed typical myopathic changes including low-amplitude and short-duration action potentials. Nerve conduction studies indicated normal motor conduction velocities. The muscle biopsies showed dystrophic-like changes such as variability in myofiber diameters, atrophic and hypertrophic fibers, in addition, to increase in adipose and fibrous tissue.

Informed consent was taken from the family and the university approved the study ethically. 

In immunohistochemical studies, the complete absence of all four sarcoglycans was indicated. In order to find underlying genetic cause, genomic DNA was extracted from blood samples of the patients using the standard salting out method after informed consent. Sequence analysis was performed using NimbleGen chip capturing of LGMD related genes followed by Next Generation Sequencing (BGI-Clinical Laboratories, Shenzhen, China). Homozygous deletion of SGCB exon 2 was detected in the patients.

In order to delineate deletion boundaries, long-range PCR, and chromosomal walking technique were used.

**Fig 2:**
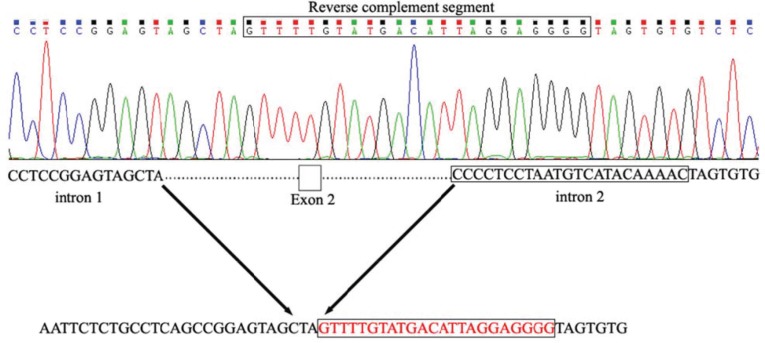
Delineation of deletion/ inversion boundaries in the patients

For this reason, genomic DNA of patient and parents was amplified with different primer pairs. Ultimately, the primer pairs 5’-TCCCCACCCTGCATTTGAA-3’ and 5’-GGCATGTTATTCTTGTTTCATTGA-3’ that correspond to adjacent introns respectively, showed the deletion boundaries. PCR conditions were as follows: an initial denaturation step at 95 °C for 2 min, 30 cycles of 95 ºC for 30 sec, 56.1 ºC for 30 sec and 72 ºC for 5 min, and a final extension step at 72 °C for 5 min. Sanger sequence analysis of the corresponding segment in the proband showed a 2082 bp deletion (NG_008891.1:g.9104_11184del, c.34-576_243+1295del, p.Gln12_Ile81del) as well as insertion of a 23 nucleotide segment which is reverse complement of a segment adjacent to the deleted region (NG_008891.1:g.11186_11208inv, c.243+1297_243+1319inv) (Figure 2). Segregation analysis showed that both parents are heterozygous for the identified mutation.

## Discussion

We have identified a novel large deletion in SGCB gene in two patients and shown that parents were heterozygous for it. Previous studies have recognized deletions of complete exon(s) of SGCG and SGCA genes, and partial duplications of exon 1 of SGCB gene (8). However, this is the first report of complete exon deletion in SGCB gene.

The existence of complete exon deletions in a certain gene has its implications in genetic diagnosis, especially for carrier detection. Therefore, such probability should be considered in genetic counseling of LGMD patients.

In addition, the patients presented above had no more severe phenotype compared with the cases presented in the literature before despite the different nature of mutation. For instance, a missense mutation in exon 3, encoding for the proximal extracellular domain caused a severe a Duchenne-like phenotype. 

Missense mutations affecting this domain would lead to the instability of the entire sarcoglycan complex resulting in severe phenotypes as seen in nonsense mutations (9). Additionally, although shown cardiomyopathy in LGMD type 2E patients (10), our patients did not have any manifestations of cardiomyopathy at the age of 12.

**In conclusion, **Future studies would help in establishment of genotype-phenotype correlation in LGMD patients that is of special importance in genetic counseling as well as prenatal diagnosis.
